# COG4 mutation in Saul-Wilson syndrome selectively affects secretion of proteins involved in chondrogenesis in chondrocyte-like cells

**DOI:** 10.3389/fcell.2022.979096

**Published:** 2022-10-28

**Authors:** Zhi-Jie Xia, Sonal Mahajan, Earnest James Paul Daniel, Bobby G. Ng, Mayank Saraswat, Alexandre Rosa Campos, Rabi Murad, Miao He, Hudson H. Freeze

**Affiliations:** ^1^ Human Genetics Program, Sanford Burnham Prebys Medical Discovery Institute, La Jolla, CA, United States; ^2^ Department of Pathology and Laboratory Medicine, Children’s Hospital of Philadelphia, Philadelphia, PA, United States; ^3^ Immunity and Pathogenesis Program, Sanford Burnham Prebys Medical Discovery Institute, La Jolla, CA, United States; ^4^ Proteomics Facility, Sanford Burnham Prebys Medical Discovery Institute, La Jolla, CA, United States; ^5^ Bioinformatics Core, Sanford Burnham Prebys Medical Discovery Institute, La Jolla, CA, United States

**Keywords:** COG4, Saul-Wilson Syndrome, 3D culture, secretome, mass-spectrometry, N-glycan

## Abstract

Saul-Wilson syndrome is a rare skeletal dysplasia caused by a heterozygous mutation in COG4 (p.G516R). Our previous study showed that this mutation affected glycosylation of proteoglycans and disturbed chondrocyte elongation and intercalation in zebrafish embryos expressing the COG4^p.G516R^ variant. How this mutation causes chondrocyte deficiencies remain unsolved. To analyze a disease-relevant cell type, COG4^p.G516R^ variant was generated by CRISPR knock-in technique in the chondrosarcoma cell line SW1353 to study chondrocyte differentiation and protein secretion. COG4^p.G516R^ cells display impaired protein trafficking and altered COG complex size, similar to SWS-derived fibroblasts. Both SW1353 and HEK293T cells carrying COG4^p.G516R^ showed very modest, cell-type dependent changes in N-glycans. Using 3D culture methods, we found that cells carrying the COG4^p.G516R^ variant made smaller spheroids and had increased apoptosis, indicating impaired *in vitro* chondrogenesis. Adding WT cells or their conditioned medium reduced cell death and increased spheroid sizes of COG4^p.G516R^ mutant cells, suggesting a deficiency in secreted matrix components. Mass spectrometry-based secretome analysis showed selectively impaired protein secretion, including MMP13 and IGFBP7 which are involved in chondrogenesis and osteogenesis. We verified reduced expression of chondrogenic differentiation markers, MMP13 and COL10A1 and delayed response to BMP2 in COG4^p.G516R^ mutant cells. Collectively, our results show that the Saul-Wilson syndrome COG4^p.G516R^ variant selectively affects the secretion of multiple proteins, especially in chondrocyte-like cells which could further cause pleiotropic defects including hampering long bone growth in SWS individuals.

## 1 Introduction

Saul-Wilson syndrome (SWS) is a rare skeletal dysplasia characterized by a distinct facial phenotype, short stature, brachydactyly, clubfoot deformities, cataracts, and microcephaly (Saul and Wilson, 1990; Ferreira et al., 2020a). Our previous study identified that SWS is caused by a heterozygous, dominant variant (p.G516R) in COG4, a Golgi-associated protein involved in protein trafficking (Ferreira et al., 2018). The COG4^p.G516R^ variant did not show a reduced protein level, in contrast to loss-of-function mutations in COG4, which causes COG4-deficient congenital disorder of glycosylation (CDG) (Reynders et al., 2009; Ng et al., 2011). COG4-CDG is usually lethal and characterized by neurological deficiencies, microcephaly, and impaired N-glycosylation. Although SWS individuals also show microcephaly, their N-glycans and neurological features appear normal (Ferreira et al., 2018; Ferreira, 2020; Ferreira et al., 2020b).

Zebrafish models for both COG4-CDG and COG4-SWS have been reported and they all display small body length, abnormal pectoral fins, and abnormal chondrocyte stacking (Ferreira et al., 2018; Clement et al., 2019; Xia et al., 2021)**.** But COG4-null zebrafish exhibit more severely impaired proteoglycan synthesis based on reduced Alcian blue staining of the jaw compared to COG4^p.G516R^ zebrafish. Our previous work also revealed that the COG4^p.G516R^ variant disturbed WNT4 signaling at the embryonic stage in zebrafish development. These differences likely indicate distinctive mechanisms for the dominant verses recessive COG4 disorders (Xia et al., 2021).

COG4 is a subunit of the conserved oligomeric Golgi (COG) complex which belongs to complexes associated with tethering containing helical rods (CATCHR) family (Yu and Hughson, 2010; Blackburn et al., 2019). CATCHR complexes play critical roles in vesicle tethering, intra-Golgi trafficking, Golgi homeostasis, and membrane trafficking (Lees et al., 2010; Blackburn et al., 2019; Adusumalli et al., 2021). It is not surprising that the COG4^p.G516R^ variant could disturb the secretion of multiple proteins. Our previous findings of impaired decorin glycosylation and glypican turnover prompted a more thorough study of protein secretion in a suitable cell line carrying the COG4^p.G516R^ variant (Ferreira et al., 2018; Xia et al., 2021). Sumya et al. have shown increased secretion of intracellular protein SIL1 and ERGIC53 in COG4-G516R RPE1 cells compared to WT. But a more relevant, bone-related cell type might reveal different candidates in a secretome study, since mutant RPE1 cells (p.G516R and p. R729W) did not phenocopy patient fibroblasts (Sumya et al., 2021). Abnormal chondrocyte intercalation and elongation in SWS zebrafish prompted us to choose chondrocyte-like cells to examine the overall protein secretion affected by COG4^p.G516R^ variant.

Chondrocytes secret extracellular matrix (ECM) proteins, growth factors, and enzymes which further regulate ECM synthesis (Melrose et al., 2016; Chijimatsu and Saito, 2019; Chen et al., 2021). During endochondral ossification, chondrocytes undergo a series of differentiation steps to form the growth plate with the support of the ECM (Harley, 1988; Yang et al., 2014). Three-dimensional (3D) cell culture is an emerging technology that allows a more physiological expansion and differentiation of cells compared to cultivation on conventional 2D systems (Benya and Shaffer, 1982; Wang et al., 2009; Studer et al., 2012). Pellet culture is a scaffold-free 3D culture form, which is commonly used to stabilize the chondrogenic potential of *in vitro* cultured chondrocytes (Caron et al., 2012; Grigull et al., 2020). Moreover, studies of using tumor cells showed that spheroids formed in *in vitro* 3D models exhibit physiologically relevant cell-cell and cell-matrix interactions, gene expression and signaling pathway profiles, bridging the gap between 2D culture models and *in vivo* whole animal systems (Nath and Devi, 2016).

Therefore, this paper aims to utilize cell spheroids obtained by 3D culture methods as a quantitative biomarker and mass spectrometry (MS)-based secretome analysis to investigate the specific changes caused by COG4^p.G516R^ variant compared to WT and COG4-KO in chondrocyte-like cells to extend the picture beyond skin fibroblasts.

## 2 Results

### 2.1 Chondrosarcoma COG4^p.G516R^ and COG4-null cells retain key features of SWS- and COG4-CDG-derived fibroblasts

**FIGURE 1:**
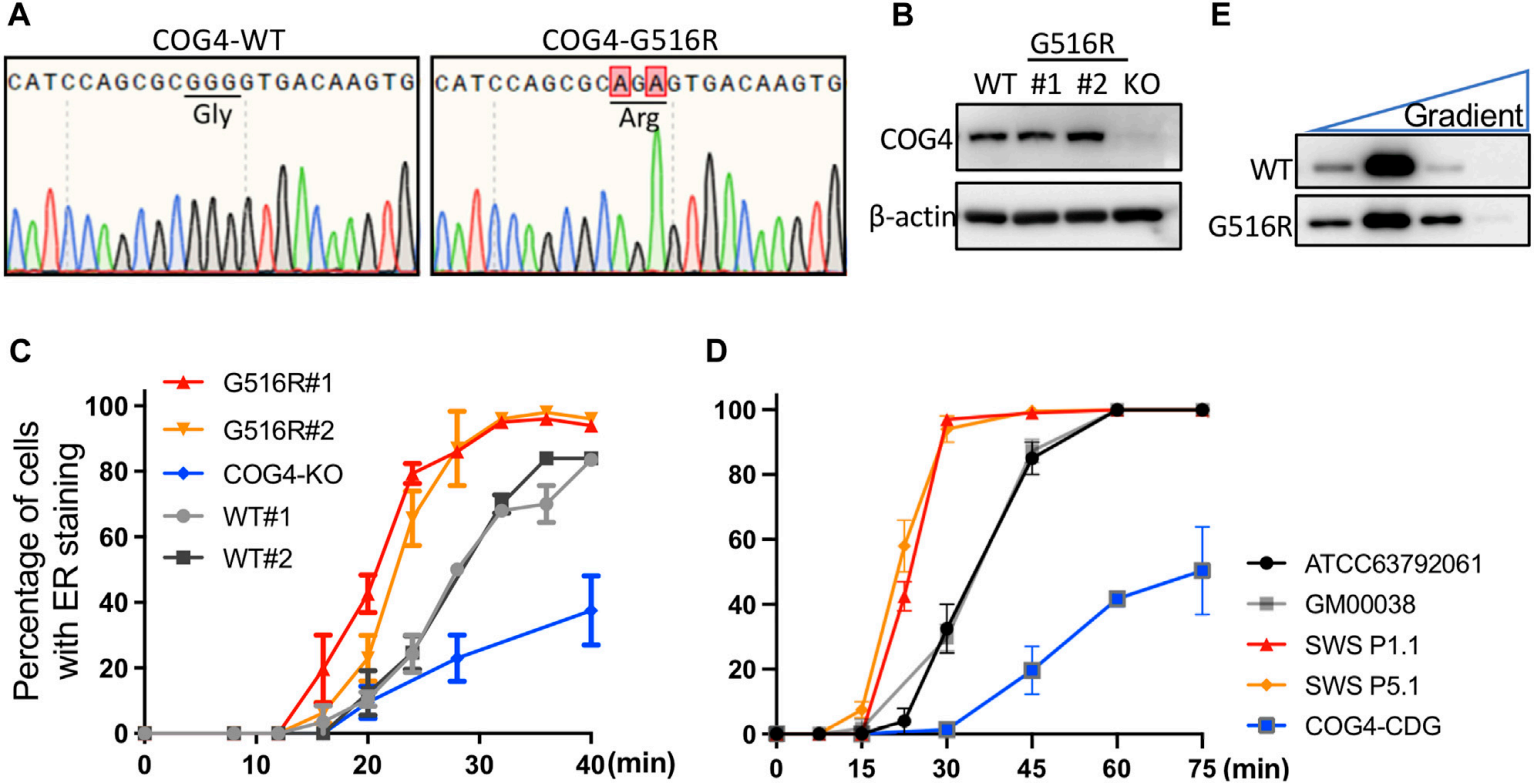
Generated COG4^p.G516R^ and COG4-KO cells show similar features as SWS- and COG4-CDG-derived fibroblasts. **(A)** Sequencing verification of COG4^p.G516R^ CRISPR knock-in in chondrosarcoma SW1353 cells. **(B)** Western blotting verification of COG4^p.G516R^ and COG4-KO SW1353 cells. **(C)** BFA-induced retrograde trafficking in COG4^p.G516R^ and COG4-KO cells compared to WT SW1353 cells. **(D)** BFA-induced retrograde trafficking in control, SWS and CDG-COG4 fibroblasts. GM00038 and COG4-CDG were performed in this study. ATCC63792061, SWS P1.1 and P5.1 were from published data (Ferreira et al., 2018). Data were presented as mean with standard error (SD). **(E)** COG complex shifts to heavier fraction in glycerol gradient in SW1353 cells expressing COG4^p.G516R^ variant. Western, trafficking and gradient experiments were performed in triplicate with similar results.

Since SWS is a skeletal disorder, the chondrocyte-like cell line SW1353 was chosen to generate COG4^p.G516R^ and COG4 knock-out (KO) cells by using CRISPR technology. Generated cell lines were verified by sequencing and Western blots (Figures 1A,B). The protein level of COG4^p.G516R^ variant did not decrease, while COG4-KO cells displayed a total loss of COG4, as expected (Figure 1B). We further evaluated whether these mutant cells mimic the features of patient fibroblasts. One of the most significant changes in SWS-derived fibroblasts was altered protein trafficking between the ER and Golgi (Reynders et al., 2009; Ferreira et al., 2018). COG4^p.G516R^ cells show accelerated Brefeldin A (BFA)-induced retrograde transport as seen previously in SWS-derived fibroblasts. In contrast, COG4-KO cells, show decreased retrograde transport as seen in COG4-CDG patient fibroblasts [Figures 1C,D (Ferreira et al., 2018)]. Another change seen in fibroblasts from SWS individuals was an apparently enlarged COG complex hydrodynamic volume based on glycerol gradient ultracentrifugation. We observed an evident shift of COG complex to the heavier fraction in SW1353 cells carrying COG4^p.G516R^ mutation (Figure 1E) as seen in SWS individual’s fibroblasts.

### 2.2 COG4^p.G516R^ and COG4-null cells show differently impaired N-glycans in different cell types

**FIGURE 2:**
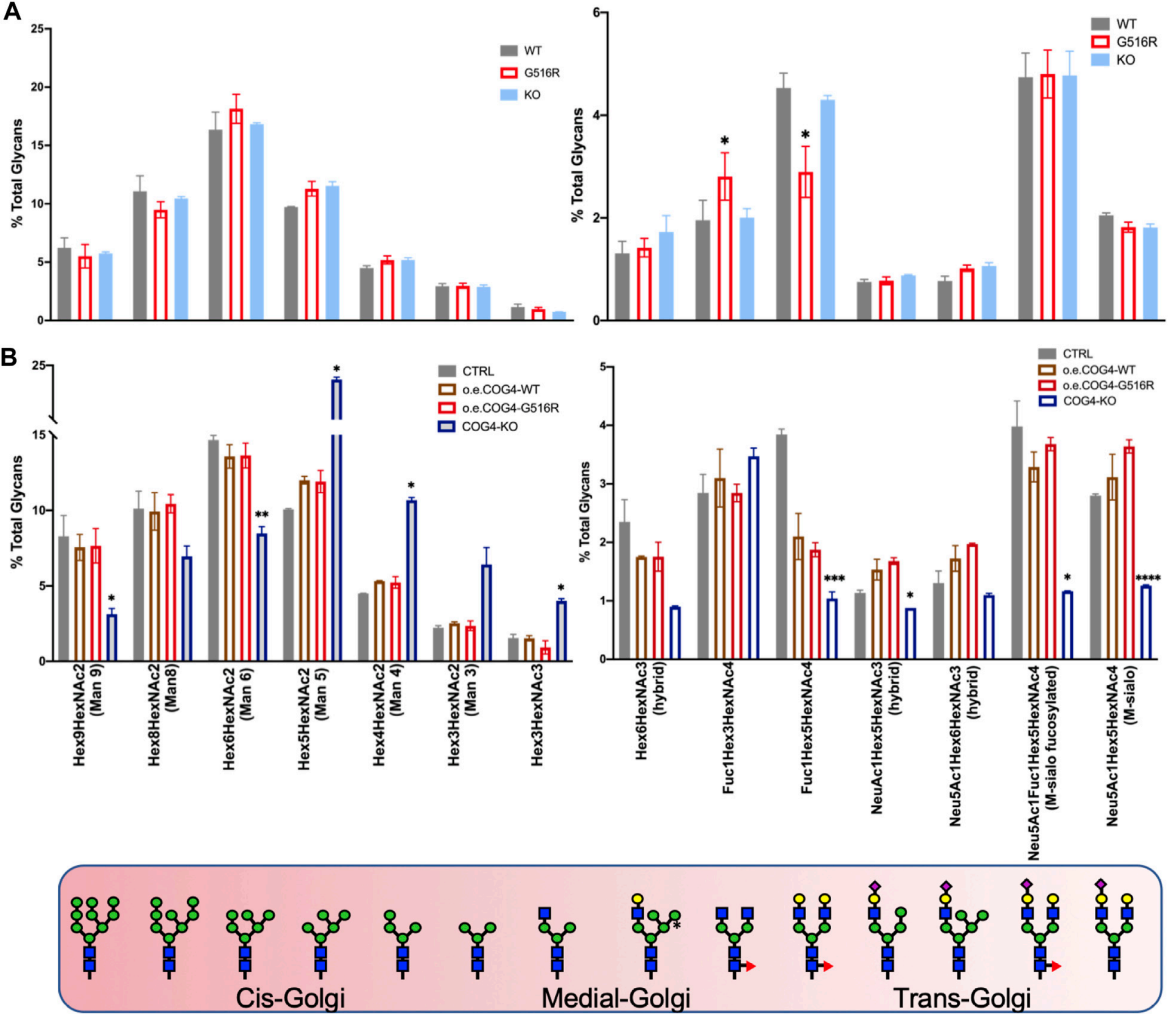
N-glycan analysis of SW1353 and HEK293T cells carrying COG4 mutations. **(A)** N-glycans of SW1353 COG4^p.G516R^ and COG4-KO cells compared to WT SW1353 cells. **(B)** N-glycans of HEK293T cells overexpressing WT-COG4-Myc or G516R-COG4-Myc or COG4-KO compared to control HEK293T cells. The different Golgi cisterna tags show the location of indicated glycans. Data were presented as bar graph with mean and SD. Experiments were performed in duplicate with similar results. Unpaired two-tailed *t*-test was used. ****, *p* < 0.001; ***, *p* < 0.005; **, *p* < 0.01; *, *p* < 0.05.

Our initial studies did not find significant N-glycan changes in SWS-derived fibroblasts (Ferreira et al., 2018). Here we compared N-glycans in different cell types carrying COG4 mutations to examine possible cell-type dependent changes. Chondrosarcoma cells carrying COG4^p.G516R^ variant and COG4-KO exhibited subtle differences in N-glycans (Figure 2A). There were two specific changes in COG4^p.G516R^ cells showing increased Fuc1HexHexNAc3 and decreased Fuc1Hex5HexNAc4, which were synthesized in medial- and trans-Golgi respectively (Figure 2A, right panel). For comparison purposes, we also generated stable cell lines expressing WT COG4 and COG- COG4^p.G516R^ under pCMV6 promotor in HEK293T COG4-KO cells (a gift from Prof. Lupashin (Blackburn and Lupashin, 2016)), respectively and verified the COG4 expression by Western (Supplementary Figure S1). HEK293T cells expressing COG4^p.G516R^ mutation did not show specific changes in N-glycans (Figure 2B). As expected, COG4-KO HEK293T cells show dramatic changes in multiple N-glycans including decreased sialylated and galactosylated glycans, specifically Mono-sialo, Mono-sialo fucosylated and Fuc1Hex5HexNAc4 (Asialo fucosylated) glycans (Figure 2B, right panel). Interestingly, COG4-KO HEK293T cells displayed altered abundance of some high mannose N-glycans including significant decrease in Man6 and Man9 and increase in Man4 and Man5 (Figure 2B, left panel) which were not seen in COG4-CDG patient serum samples (Reynders et al., 2009). These changes in N-glycans of COG4-KO cells suggested that COG4 KO affect multiple stages of N-glycan processing in the whole Golgi and preferentially, in the early processing steps in the cis-medial Golgi. Overall, N-glycosylation changes in COG-COG4^p.G516R^ are extremely subtle and cell-type dependent in comparison to WT.

### 2.3 Pellet culture of COG4^p.G516R^ show reduced spheroid size and increased apoptosis compared to WT and COG4-null cells

**FIGURE 3:**
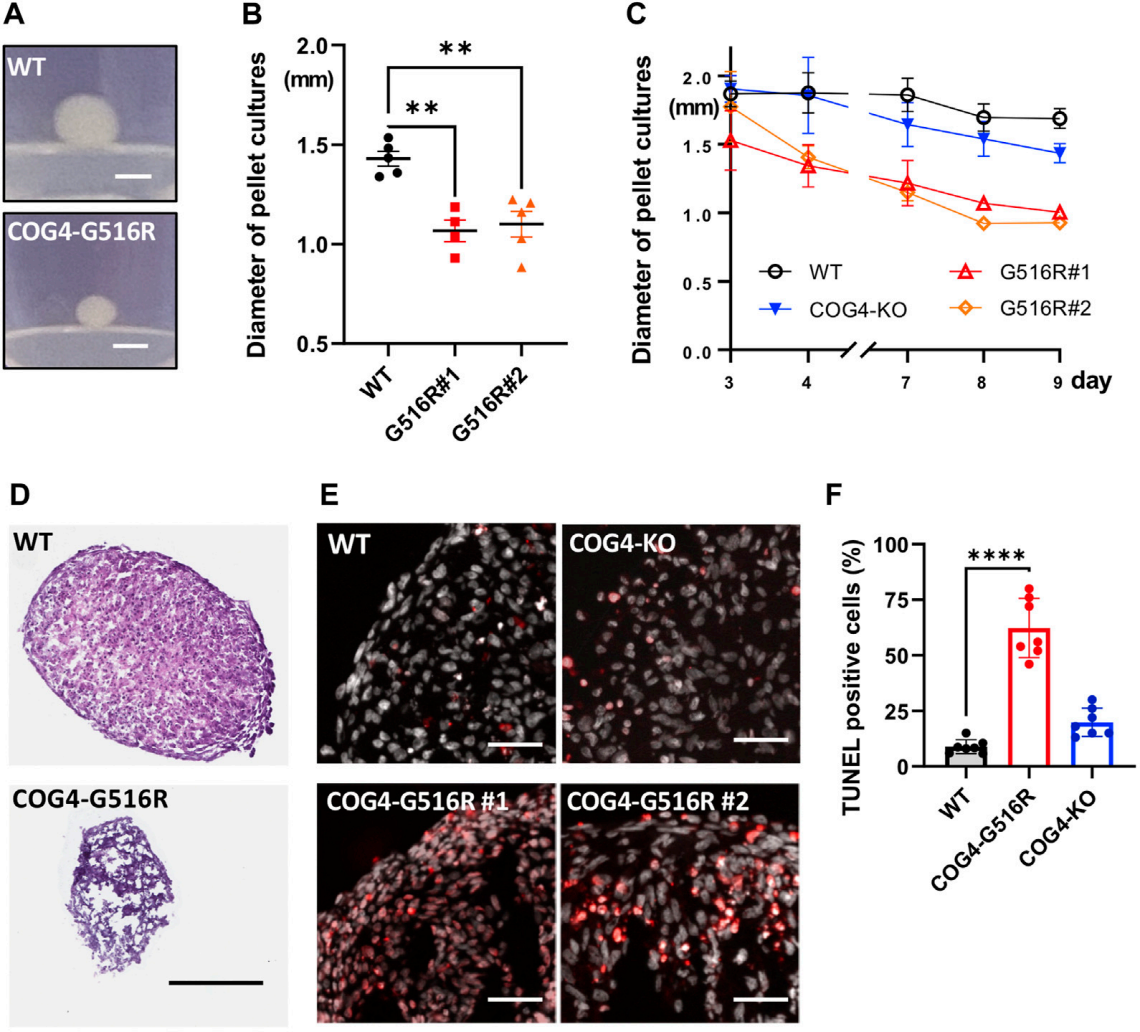
Chondrosarcoma cells carrying *COG4* ^*p.G516R*^ mutation show different features compared to WT and COG4-KO cells. **(A)** Representative images of aggregates formed by WT or COG4^p.G516R^ cells in pellet culture. Bar, 1 mm. **(B)** Diameter measurement of the spheroids on Day 10 (*n* = 5). Data were presented as scatter dot plot with mean and SEM. **(C)** Diameter measurement of the spheroids over 9 days (*n* = 5). Data were presented as mean with SD. **(D)** Representative images of H&E staining of spheroids on Day 6. Bar, 300 µm. **(E)** Representative images of TUNEL assay on Day 4. Bar, 50 µm. **(F)** Statistical analysis of **(E)** (*n* = 7) Data were presented as mean with SD. All experiments were performed in duplicate with similar results. Unpaired two-tailed *t*-test was used. ****, *p* < 0.001; **, *p* < 0.01.

A scaffold-free 3D culture format, pellet culture, was used to examine the *in vitro* chondrogenic differentiation of chondrosarcoma SW1353 cells. COG4^p.G516R^ showed significantly reduced spheroid sizes on Day 10 compared to WT and COG4-KO cells (Figures 3A–C). Hematoxylin and eosin (H&E) staining further revealed a necrotic core on day 6 in COG4^p.G516R^ cells (Figure 3D). Increased apoptosis was exclusively seen in COG4^p.G516R^ cells starting at Day 4 (Figures 3E,F), which probably explains the reduced aggregate size**.** We also examined the aggregate formation of control and SWS-derived fibroblasts using pellet culture. However, we did not see differences in terms of aggregate sizes and Alcian blue staining (Supplementary Figures S2A–S2C). H&E staining did not show significant differences except for slightly reduced fringe cells in SWS and COG4-CDG cells (Supplementary Figure S2D). Mildly increased apoptosis was also seen in SWS-derived fibroblasts, similar to COG4^p.G516R^ chondrosarcoma cells (Figure 3E).

### 2.4 Mixed cultures of COG4^p.G516R^ with WT cells and medium supplement rescue spheroid sizes and cell death

**FIGURE 4:**
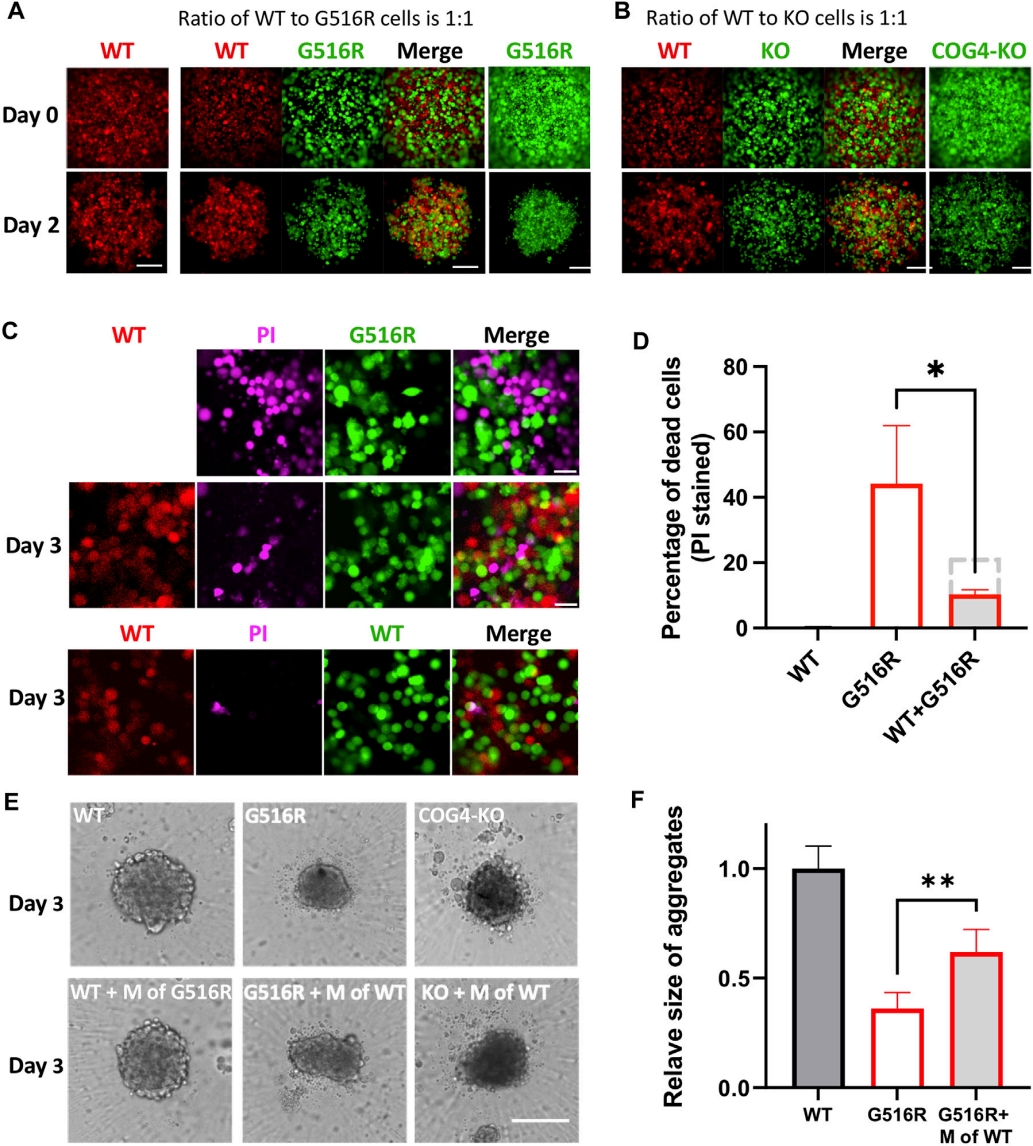
Mixed cultures of COG4^p.G516R^ and COG4-KO cells with WT cells and conditioned medium supplement testing. **(A)** Representative confocal images on Day 0 and Day 2 of mixed cultures of COG4^p.G516R^ and COG4-KO cells with WT cells. Left column, WT cells expressing RFP only. Middle panels, mixed culture of COG4^p.G516R^ (GFP labeled) and WT (RFP labeled). Right column, COG4^p.G516R^ cells expressing GFP only. Bar, 200 µm. **(B)** Left panels, mixed culture of COG4-KO (GFP labeled) and WT (RFP labeled). Right column, COG4-KO cells only. **(C)** Top panel, PI staining of COG4^p.G516R^ (GFP labeled) only. Middle panel, PI staining of mixed culture of COG4^p.G516R^ (GFP labeled) and WT (RFP labeled). Bottom panel, PI staining of mixed culture of WT-RFP and WT-GFP. Bar 50 µm. **(D)** Statistical analysis of **(C)** (*n* = 3). The dashed grey line indicates predicted PI staining cells if no interaction presents between WT and G516R cells. **(E)** Conditioned medium of WT as a supplement for spheroid formation in ULA plate. Top panel, WT, COG4^p.G516R^, and COG4-KO cells only. Bottom panel, WT cells supplemented with conditioned medium of COG4^p.G516R^ and COG4^p.G516R^ and COG4-KO cells supplemented with conditioned medium of WT on Day 3. Bar, 200 µm. **(F)** Statistical analysis of middle panel **(E)**, area measurements. All experiments were performed in duplicate with similar results. Unpaired two-tailed *t*-test was used. **, *p* < 0.01; *, *p* < 0.05.

To study the interplay between WT and mutant cells, we made mixed cultures in 384-well ultra-low attachment (ULA) plates and evaluated the effect on the spheroid formation. We first fluorescently labeled WT, COG4^p.G516R^, and COG4-KO chondrosarcoma cells by expressing cytosolic RFP or GFP. Then WT and mutant cells were either cultured alone or mixed in a one-to-one ratio (Figure 4A). When cultured alone in ULA plates, COG4^p.G516R^ cells showed reduced size on Day 2 (Figure 4A, right bottom) compared to WT (Figure 4A, left bottom) with the same number of cells. Interestingly, in mixed cultures of WT and COG4^p.G516R^ cells, the aggregate size was visibly increased (Figure 4A, middle panel). We did not see changes in mixed culture of WT and COG4-KO cells (Figure 4B). We further examined the cell viability by Propidium Iodide (PI) staining and found that mixed culture with WT could significantly reduce cell death of COG4^p.G516R^ cells (Figures 4C,D). Interestingly, the conditioned medium collected from WT cells could partially rescue the spheroid sizes of COG4^p.G516R^ cells by about 25% (Figures 4E,F). This effect was further confirmed by checking the aggregate formation on poly-lysine coated plates (Supplementary Figure S3). In both systems, adding conditioned medium from COG4^p.G516R^ cells to WT cultures did not decrease spheroid size or cause cell death, showing that the WT medium was providing factors that were deficient in the medium of COG4^p.G516R^ cells.

### 2.5 MS-based secretome showed selectively impaired protein secretion in COG4-mutant cells

**FIGURE 5:**
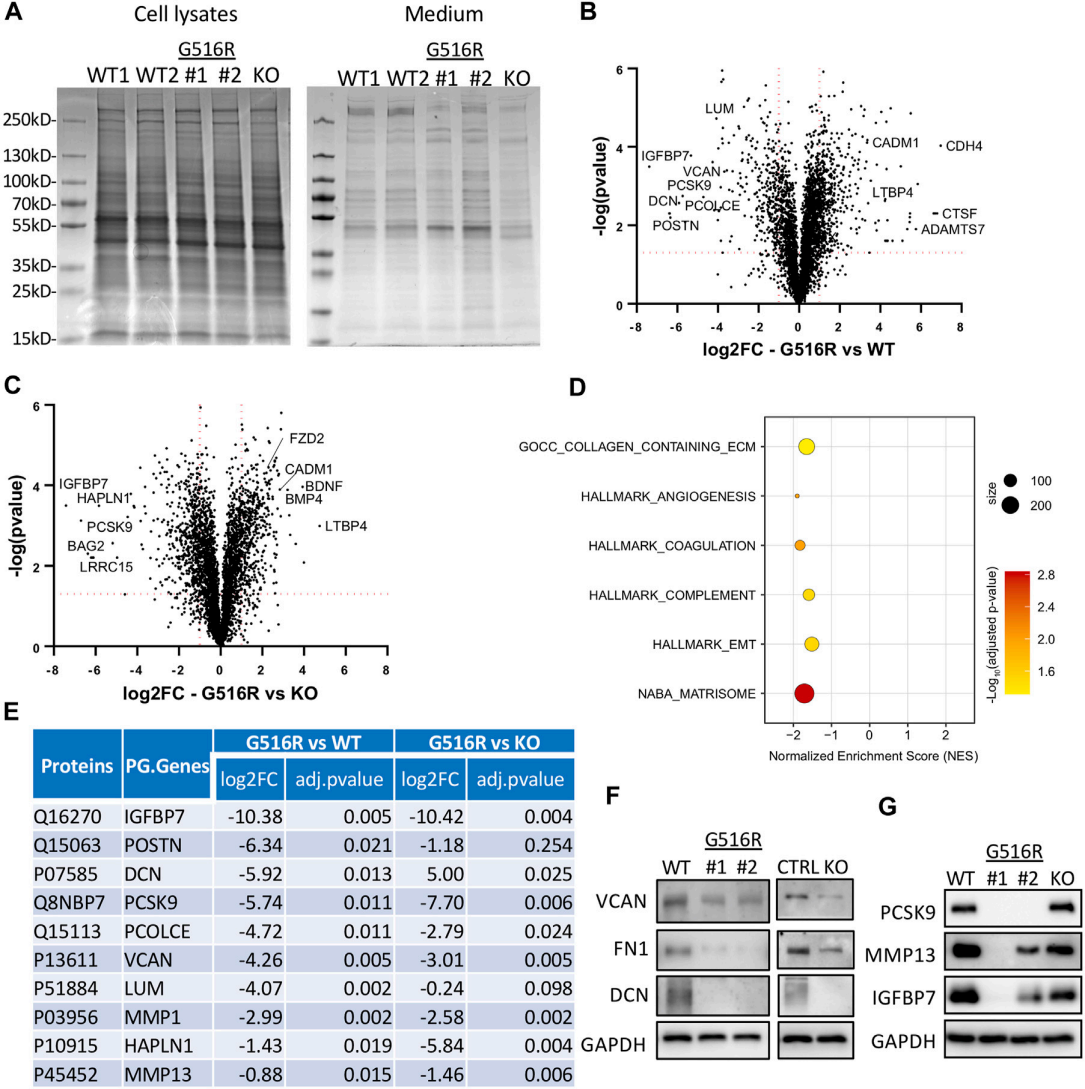
Secretome analysis of WT, COG4^p.G516R^, and COG4-KO chondrosarcoma cells. **(A)** Coomassie blue staining of proteins in cell lysates and conditioned medium from COG4^p.G516R^ clones #1, #2, WT controls and COG4 KO cells. **(B)** Volcano plot of differentially secreted proteins between COG4^p.G516R^ and WT. **(C)** Volcano plot of differentially secreted proteins between COG4^p.G516R^ and COG4-KO cells. A few top candidates confirmed by pathway analysis were labeled respectively. **(D)** Dot plot showing enriched pathways from Gene Set Enrichment Analysis with the Normalized Enrichment Score (NES) shown on the *X*-axis. The size of the dots represents the number of genes in the significant Data Set list associated with the GO term and the color of the dots represent the adjusted p- values. **(E)** Table showing a list of top 10 candidates which were decreased in secretions of COG4^p.G516R^ with log 2 (Fold Change) and adjusted *p*-value shown for COG4^p.G516R^ vs. WT and COG4^p.G516R^ vs. COG4-KO. The number of peptides is equal to or more than two for all the candidates shown in table. **(F)** Western blotting of top candidates in medium decreased in both COG4^p.G516R^ and COG4-KO cells compared to WT. GAPDH was from cell lysates with comparable amount as loading control. **(G)** Western blotting of top candidates only changed in COG4^p.G516R^ compared to WT and COG4-KO cells. All experiments were performed in duplicate with similar results.

Prompted by the WT medium complementation experiments, we examined the proteins in the conditioned medium by Coomassie-blue staining after separating proteins in polyacrylamide gels. We observed some altered protein bands in chondrosarcoma COG4 mutant cells which were not seen in cell lysates (Figure 5A). MS-based proteomics was further performed to identify all proteins in the conditioned medium. The comparisons of COG4^p.G516R^ vs. WT and COG4^p.G516R^ vs. KO were displayed as Volcano plots (Figures 5B,C). To determine the functional meaning of the differentially regulated secreted proteins, we performed Gene Set Enrichment Analysis (GSEA) by using different Human Gene Sets in Molecular Signatures Database. We found a significant over-representation of categories mainly related to extracellular matrix and some others, with few top candidates as “Naba_Matrisome”, “GOCC_Collagen_Containing_Extracellular_Matrix”, “Angiogenesis”, “Complement”, “Coagulation” and “Epithelial_Mesenchymal_Transition” (Figure 5D). We see about 84 proteins with significantly decreased measurement and 48 proteins with significantly increased measurement, belonging to “Naba Matrisome”. The altered Matrisome proteins includes various proteoglycans (Decorin, Versican, Lumican, hyaluronan and proteoglycan link protein 1), glycoproteins (IGFBPs, LTBPs, Fibulins, laminins, PCOLCE, POSTN), various classes of collagens, ECM Regulators (ADAM metallopeptidases, MMPs, serpin peptidase inhibitor, cathepsins), ECM-affiliated Proteins (Glypicans, syndecans, annexins) and secreted factors (BMPs, WNT proteins, growth factors, chemokines). Many of these deficient Matrisome proteins are common regulators of other affected pathways. A list of top 10 candidates which were decreased in secretions of COG4^p.G516R^ as compared to WT is shown in Figure 5E. Our secretome data showed that some normally secreted proteins were deficient in both COG4^p.G516R^ and COG4-KO cells including fibronectin (FN1) and some proteoglycans (versican and decorin), which we further verified in western blots (Figure 5F). Interestingly, we also found several proteins that were exclusively missing only in COG4^p.G516R^ cells (Figure 5E). Several top candidates were verified by Western blotting, including matrix metallopeptidase 13 (MMP13), insulin-like growth factor binding protein 7 (IGFBP7), and proprotein convertase subtilisin/kexin type 9 serine protease (PCSK9) (Figure 5G), confirming the MS findings. A few ER stress markers were also tested because of reduced secretion, although none of them were top candidates in our list. Western blotting of these markers did not show increased protein level indicating no ER stress in COG4^p.G516R^ cells (Supplementary Figure S4). Conditioned medium from control and SWS patient fibroblasts were also analyzed by MS. However, fibroblasts gave many fewer candidates than chondrosarcoma cells (Supplementary Figure S5), indicating the importance of choosing a relevant cell type to study skeletal disorders.

### 2.6 Chondrogenic differentiation was impaired exclusively in COG4^p.G516R^ cells

**FIGURE 6:**
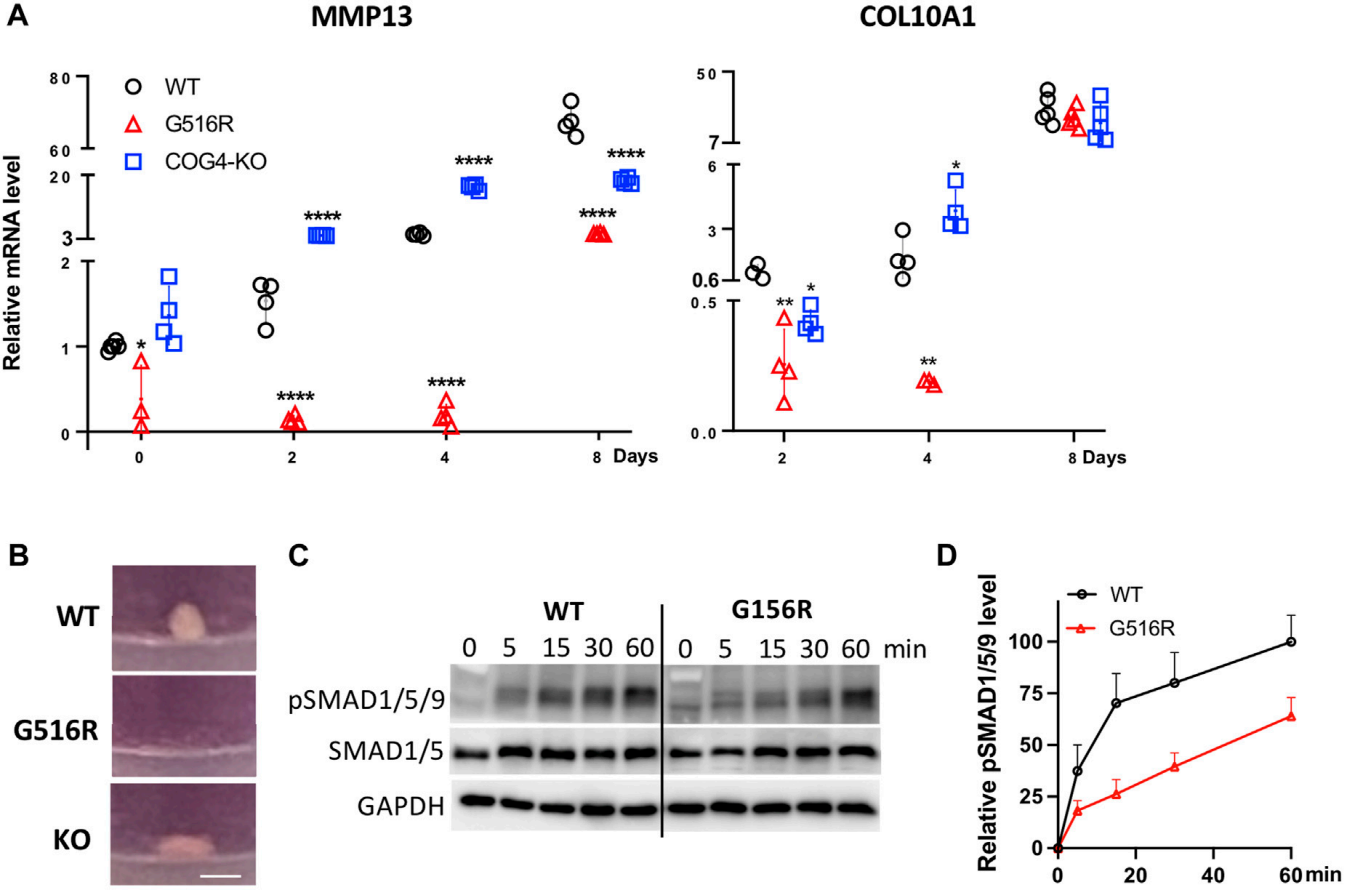
COG4^p.G516R^ chondrosarcoma cells show impaired chondrogenic differentiation. **(A)** mRNA level of chondrogenic markers, MMP13 and COL10A1. Data were presented as scatter dot plot with mean and SD. Unpaired two-tailed *t*-test was used. ****, *p* < 0.001; **, *p* < 0.01; *, *p* < 0.05. **(B)** Representative images of spheroid/pellet with the presence of 10 ng/ml BMP2 for 14 days. Bar, 1 mm. **(C)** Time course of BMP2-induced phosphorylation of Smad1/5/8. Monolayer cultures were stimulated with 10 ng/ml BMP2, and cells were lysed after indicated time of incubation. **(D)** Statistical analysis of pSMAD1/5/9 in **(C)**. All experiments were performed in triplicate with similar results.

MMP13 and proteoglycans are critical for chondrocyte differentiation, especially from proliferating chondrocytes to hypertrophic chondrocytes (Studer et al., 2012; Komori, 2022). Prompted by the MS data, we further examined the mRNA level of differentiation markers, MMP13 and COL10A1 of aggregates in pellet culture treated with TGFβ3. We found that COG4^p.G516R^ cells showed profoundly low levels of MMP13 on Day 0–4 and COL10A1 on Day 2–4 (Figure 6A). We could not detect COL10A1 expression on Day 0. After 8 days of treatment with TGFβ3, mRNA level of MMP13 in COG4^p.G516R^ cells was still significantly lower than WT and COG4-KO cells, while COL10A1 level could catch up on Day 8 (Figure 6A). Using fibroblasts, we did not see impaired COL10A1 and MMP13 mRNA levels over days in SWS patient cells compared to the control (Supplementary Figure S2F). Another chondrogenic factor, bone morphogenetic protein 2 (BMP2) was also tested using 3D and 2D cultures. In pellet culture, COG4^p.G516R^ cells could not form spheroids with the presence of BMP2 indicating disturbed chondrogenic differentiation, opposite to WT and COG4-KO cells (Figure 6B). Moreover, COG4^p.G516R^ cells exhibited lower levels of phosphorylation of Smad1/5/9 proteins in response to BMP2 than did control cells (Figures 6C,D).

## 3 Discussion

COG4 is a protein involved in protein trafficking and glycosylation, and its mutations cause both dominant and recessive human genetic disorders, SWS and COG-CDG, respectively. In this study, we aimed to choose a proper cell model to understand how the dominant COG4^p.G516R^ variant causes a primordial dwarfism. Studies in SWS-derived fibroblasts and HEK293T cells provide confidence that the molecular characteristics and trafficking abnormalities are maintained and are an inherent property of the mutated protein, but chondrocyte-like SW1353 has several advantages. SW1353 cells carrying COG4^p.G516R^ mutation not only manifest the distinctive defects seen in SWS-derived fibroblasts, but also synthesize a more disease-relevant phenotype displaying changes of ECM protein secretion, indicating a suitable cell model to study SWS.

We previously reported that N-glycans in SWS patient serum samples and fibroblasts were not significantly changed compared to controls (Ferreira et al., 2018). In this study, we applied higher resolution MS technology, but found only very subtle changes in a few N-glycans in chondrosarcoma cells carrying COG4^p.G516R^ mutation, typical of SWS patient samples. Similarly, essentially no N-glycan changes were seen in HEK293T cells expressing COG4^p.G516R^ variant. On the other hand, COG4-null cells showed severe changes in N-glycans. It has been reported that decreased sialylation of N-glycans was a common observation in COG-CDG patient serum samples with COG4-CDG showing the least severely affected (Reynders et al., 2009). Our study confirmed this observation, and we also found dramatic changes in the abundance of high mannose structures, suggesting that COG4 loss impacts the whole Golgi, but primarily the initial mannose processing of N-glycans in the cis-medial Golgi in HEK293T cells. The dramatic N-glycans changes in HEK 293T COG4-KO cells compared to the SW1353 COG4-KO cells further shows cell-type specific impact of COG4 KO on N-glycosylation. One possible explanation could be that the glycosylation is affected by multiple factors that may vary with cell type including the integrity of the Golgi peripheral membrane proteins, the Golgi membrane dynamics, growth factor signaling, and cellular stress in addition to the primary spectrum of glycosyltransferases (Stanley, 2011) and knocking out COG4 may have different effect on trafficking of any of these glycosylation components in different cell types.

3D culture models have gained growing acceptance as *in vitro* tools to study bone diseases (Salamanna et al., 2016; Langhans, 2018; Bicer et al., 2021). In the presence of TGFβ3 and BMP2, chondrocyte pellets proliferate, producing hypertrophic chondrocytes, and the extraordinary cell volume increase during hypertrophy is accompanied by an up-regulation of COL10A1 and MMP13 (Studer et al., 2012; Yang et al., 2014). The low levels of COL10A1 and MMP13 detected in SW1353 COG4^p.G516R^ spheroids imply impaired hypertrophic differentiation which could further affect long bone growth. In addition to forming no spheroids, impaired response to BMP2 in COG4^p.G516R^ cells was confirmed by the decreased phosphorylation of SMAD1/5/9 which is required for endochondral bone formation (Retting et al., 2009). ULA plate blocks cell attachment and causes cells in suspension to aggregate into visible spheroids. Altered spheroid size and increased cell death rate verified in 384-well ULA plates also provide promising quantitative markers in high-throughput drug screening for SWS in the future.

Secretome data confirmed the reduced protein level of MMP13 and COL10A1 and revealed more changes in protein secretion in COG4 mutant cells. GSEA of COG4^p.G516R^ vs. WT data showed the most significant enrichment with “Naba Matrisome” geneset, which is a depository of not only core ECM proteins but also ECM-affiliated proteins, ECM regulators and secreted factors, many of which are known to bind to ECM (Naba et al., 2016). In both COG4^p.G516R^ and COG4-KO chondrosarcoma cells, secretion of some proteoglycans was significantly altered. It is well established that proteoglycans in the ECM play critical roles in skeletal development by interacting with secreted growth factors (Koosha and Eames, 2022). The reduced ECM proteoglycans in both clonal cell lines might explain the common skeletal deficiencies seen in both SWS and COG4-CDG patients. Interesting, we identified some candidates that are only increased or decreased in COG4^p.G516R^ cells. Our top candidates include decreased IGFBP7, an important growth factor involved in osteoblastic reprogramming of fibroblasts (Lu et al., 2020; Koosha and Eames, 2022), COL1A2 and HAPLN1 which are important components of ECM. COL1A2 deficiencies cause Osteogenesis Imperfecta (OI) which manifests overlapping features with SWS as blue sclerae, bone fragility and hearing loss (Ferreira, 2020). Interestingly, we also found exclusively elevated components in the medium of COG4^p.G516R^ cells, including WNT signaling receptor FZD2, glypican 1 (GPC1), and LTBP4. These results reinforce the critical roles of WNT signaling pathway in chondrogenic hypertrophy and SWS pathogenesis (Studer et al., 2012; Xia et al., 2021). We are aware that FZD2 and GPC1 are membrane proteins instead of secreted or ECM proteins. The possible reason of the detection of GPC1 could be proteoglycan shedding which is considered a powerful post-translational modification (Niehrs, 2012; Xia et al., 2021). Also, the detection of some cytoplasmic proteins in the chondrocyte secretome using proteomic techniques has been reported previously and one of the potential explanations is considered as secretion of ECM-derived vesicles by chondrocytes. These vesicles contain enzymes and substrates necessary for mineral formation and believed to be one of the possible reasons for the cytoplasmic/membrane proteins, such as annexins, in the secretome of the chondrocytes (Sanchez et al., 2017). We believe SWS pathogenesis is a result of complex interplay of alterations in the matrix components, growth factors and signaling pathways, which is currently not fully understood. There is a possibility that differential secretion of some components leads to a regulation of multiple pathways. However, the mechanism behind the selectively affected protein trafficking by COG4^p.G516R^ variant and its implication in regulation of other signaling pathways is still unknown.

In summary, our findings demonstrate that chondrocyte-like cells are a suitable model to investigate the deficiencies caused by the COG4^p.G516R^ variant. Combined studies of spheroids generated by 3D culture methods and MS-based secretome analysis revealed impaired chondrogenic differentiation as a highly relevant reason contributing to SWS pathogenesis. How COG4^p.G516R^ variant selectively affects protein trafficking is our top interest and relevant studies are ongoing.

## 4 Materials and methods

### 4.1 Cell cultures

Primary fibroblasts derived from healthy controls (GM00038 and GM09503) were obtained from Coriell Institute for Medical Research (Camden, NJ). Each SWS-derived fibroblast line was obtained by the referring clinician and grown *via* a clinical lab service and then sent to us with consent through an approved IRB (Ferreira et al., 2018). SW1353 was obtained from ATCC.

Fibroblasts and SW1353 were cultured in Dulbecco’s Modified Eagle’s medium (DMEM) containing 1 g/L glucose supplemented with 10% heat-inactivated fetal bovine serum (FBS), 1X Penicillin-Streptomycin (Corning) and 200 mm L-glutamine (Corning). HEK293T cells were cultured in 4.5 g/L glucose DMEM supplemented with the same components as mentioned above.

### 4.2 Generating mutations

CRISPR knock out of COG4 in SW1353 was performed as previously described with some slight modifications (Bailey Blackburn et al., 2016; Blackburn and Lupashin, 2016). Cell Line Nucleofector™ Kit V (Lonza) was used for SW1353 transfection according to manufacturer instructions. SW1353 cells carrying COG4-G516R mutation were generated by CRISPR knock in as previously described (Ran et al., 2013; Dong et al., 2019). Primers used were: 5′-CAC​CGG​CGG​CAC​TTG​TCA​CCC​CGC​GC-3′ and 5′-AAA​CGC​GCG​GGG​TGA​CAA​GTG​CCG​CC-3'. ssODN repair template was 5′-CCT​GCC​ACC​ACC​TTC​CAG​GAC​ATC​CAG​CGC​AGA​GTG​ACA​AGT​GCC​GTG​AAC​ATC​ATG​CAC-3'. Generated COG4-G516R clones were verified by PCR with primers 5′-CCA​GTG​CTC​TGG​GGA​ATG​AAT-3′ and 5′-GGC​CAG​TCT​CCC​CTG​TAT​GT-3′ followed by sequencing.

### 4.3 Protein trafficking and complex analysis

BFA-induced protein trafficking assay and gradient ultracentrifugation were performed and analyzed as previously described (Ferreira et al., 2018).

### 4.4 N-glycan assay

N-glycans were extracted from SW1353, HEK293T and fibroblast cells as previously described (Chen et al., 2019). Briefly, N-glycans were released and derivatized using Water’s Rapifluor label, and the derivatized N-glycans were purified and concentrated using HILIC chromatography and were flow-injected into ESI-QTOF Mass Spectrometry to detect glycan intermediates by accurate Mass. An internal standard is used for quantification and percent relative abundance of glycans is reported with around 0.1% sensitivity.

### 4.5 3D cultures

In pellet culture, 4 × 10^5^ of SW1353 or fibroblast cells were centrifuged (150 g, 5 min) in a 15-ml polypropylene tube (Becton Dickinson) to form a pellet. The pellets were treated with DMEM (1 g/L glucose) containing ITS supplement, 0.2 mm L-ascorbic acid-2-phosphate, 1 mm sodium pyruvate, 0.35 mm L-proline (all Sigma-Aldrich), and 10 ng/ml TGFβ3 (Biolegend, 585802) or BMP2 (sigma H4791). Medium was changed every two or 3 days (Ullah et al., 2012; Futrega et al., 2021). Alcian blue staining and extraction of aggregates were performed as described (Wehrle et al., 2019).

In 384-well ULA plate, 3 × 10^3^ cells were seeded to each well with the medium mentioned above. Lentivirus transduction and PI staining were performed as described (Tambe et al., 2019). RFP or GFP plasmids were obtained from Addgene (#13726 and #13727). Fluorescence images were acquired using an LSM 710 confocal microscope (Zeiss, Germany).

### 4.6 H&E and TUNEL assay

H&E and TUNEL assay were performed by Histology core at Sanford Burnham Preby (SBP) following standard protocol. ApopTag^®^ Red *In Situ* Apoptosis Detection Kit (Millipore, S7165) was used for TUNEL assay.

### 4.7 Label-free proteome profiling and data analysis

SW1353 cells were grown in 10-cm dishes to 90–100% confluency, rinsed 3 times with DPBS, and one time with F12K medium (Corning™ 10025CV). Cells were incubated in F12K for 24 h, then conditioned media was collected, clarified, and concentrated using a 3 k concentrator (Amicon^®^ Ultra 3 k, Millipore). For fibroblasts, DMEM medium was used as above mentioned and incubated for 48 h before collection. Duplicate analyses were performed for each cell line.

Secreted protein profiling was performed at proteomics core at SBP. Briefly, protein samples were exchanged using 10-kDa Amicon filter (Millipore) to 8 M urea, 50 mm ammonium bicarbonate buffer. While in the filter, proteins were reduced with 5 mm tris(2-carboxyethyl) phosphine (TCEP) at 30°C for 60 min, and subsequently alkylated with 15 mm iodoacetamide (IAA) in the dark at room temperature for 30 min. The buffer was then exchanged again to 1 M urea, 50 mm ammonium bicarbonate, followed by overnight digestion with mass spec grade Trypsin/Lys-C mix. Digested peptides were then desalted in the Bravo platform using AssayMap C18 cartridges, and dried down in a SpeedVac concentrator.

Dried peptide samples were reconstituted in 2% ACN, 0.1% FA. A Proxeon EASY nanoLC system (Thermo Fisher Scientific) coupled to an Orbitrap Fusion Lumos mass spectrometer (Thermo Fisher Scientific) was used for performing proteomic analysis (LC-MS/MS). An analytical C18 Aurora column (75 µm × 250 mm, 1.6 µm particles; IonOpticks) was used to separate peptides at a flow rate of 300 nL/min using a 100-min gradient: 2%–23% B in 70 min, 23%–34% B in 20 min, 34%–98% B in 2 min, (A = FA 0.1%; B = 80% ACN: 0.1% FA). Precursors were surveyed in MS scan and settings in the Orbitrap were as follows: m/z range 350 to 1,050, 120k resolution at m/z 200, AGC 5e5 with maximum injection time of 50 ms, RF lens 30%. 3-s cycles were specified for the survey and the MS/MS scans. For DIA window size, a 10 m/z isolation window was utilized. All precursors were fragmented with 30% normalized collision energy. Scan range for MS/MS was 200–2000 m/z. AGC settings were specified as 5e5 and maximum injection time of 22 ms.

All mass spectrometry data was analyzed with Spectronaut software (Biognosys; 15.6.211,220.50606) using default settings for DierctDIA. *Homo sapiens* Uniprot protein sequence database (downloaded in February 2020) and GPM cRAP sequences (commonly known protein contaminants) were specified as sequence databases. Fully tryptic peptides with two missed cleavages were allowed with carbamidomethylation (C) as fixed modification and oxidation (M) and acetylation of protein N-terminus as a variable modification. Only the search hits corresponding to less than 1% of Q-value were accepted. MS1 intensity were used for calculating differences between the groups (*t*-test *p* < 0.05).

### 4.8 Immunoblotting

Samples were harvested using SDS lysis buffer (62.5 mm Tris-HCl, pH 6.8, 2% SDS, and 10% glycerol) supplemented with protease and phosphatase inhibitors (Sigma-Aldrich) as previously described (Ferreira et al., 2018). Equal amounts of denatured proteins were separated *via* SDS–polyacrylamide gel electrophoresis followed by transfer and antibody inoculation as described previously (Tambe et al., 2019). Antibodies used were: Smad1(Cell signaling, D59D7), Smad5 (SCBT, sc-101151), PCSK9 (SCBT, sc-515082), IGFBP7 (SCBT, sc-365293), MMP13 (SCBT, sc-515284), VCAN (Invitrogen, MA5-27638), FN1 (Abclonal, A16678), DCN (R&D, MAB143), GAPDH (Invitrogen, A5-15738), PDIA6 (Proteintech, 18233-1), GRP78 (Proteintech, 11587-1), ATF4 (SCBT, sc-390063), ATF6 (SCTB, sc-166659), pSmad159 (Cell signaling, 13820), β-actin (Cell signaling, 4970), Goat Anti-Rabbit HRP (SeraCare, 54500010), and Goat Anti-Mouse HRP (Invitrogen, 31446) diluted according to manufacturer’s instruction.

### 4.9 mRNA expression analysis

Total RNA was extracted from cell aggregates using TRIzol™ (ThermoFisher, 15596081) reagent according to manufacturer’s protocol. cDNA was synthesized using QuantiTect Reverse Transcription Kit (QIAGEN, 205311). qPCR and data analysis were performed as described previously (Tambe et al., 2019). The mRNA levels were normalized to the levels of housekeeping genes, *POLR2A and RPLP0*, and 2^−ΔΔCt^ values were calculated and compared. Three biological replicates for each point were analyzed.

### 4.10 Immunofluorescence

Fluorescence images were acquired using an LSM 510 confocal microscope (Zeiss, Germany) with a ×20 objective. Digital images were processed with Zeiss ZEN and ImageJ software.

## Data Availability

The datasets presented in this study can be found in online repositories. The names of the repository/repositories and accession number(s) can be found below: ProteomeXChange PXD036151.
