# Supplementary figures and images for: COG4 mutation in Saul-Wilson syndrome selectively affects secretion of proteins involved in chondrogenesis in chondrocyte-like cells
Source: Front Cell Dev Biol. 2022 Oct 28;10:979096. doi: 10.3389/fcell.2022.979096 (PMC9649697; doi:10.3389/fcell.2022.979096)

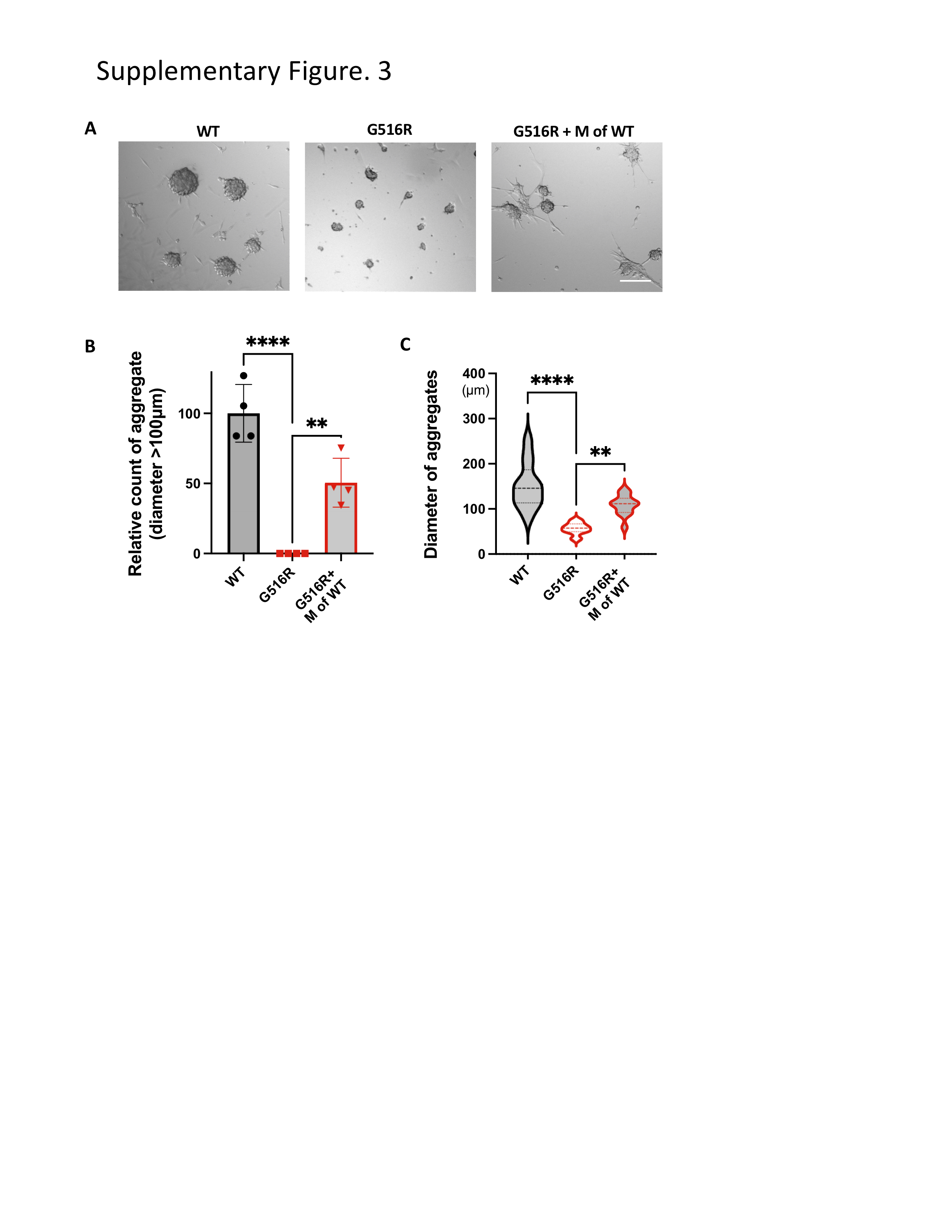

Supplement: Supplementary file 1 [file Image3.JPEG]

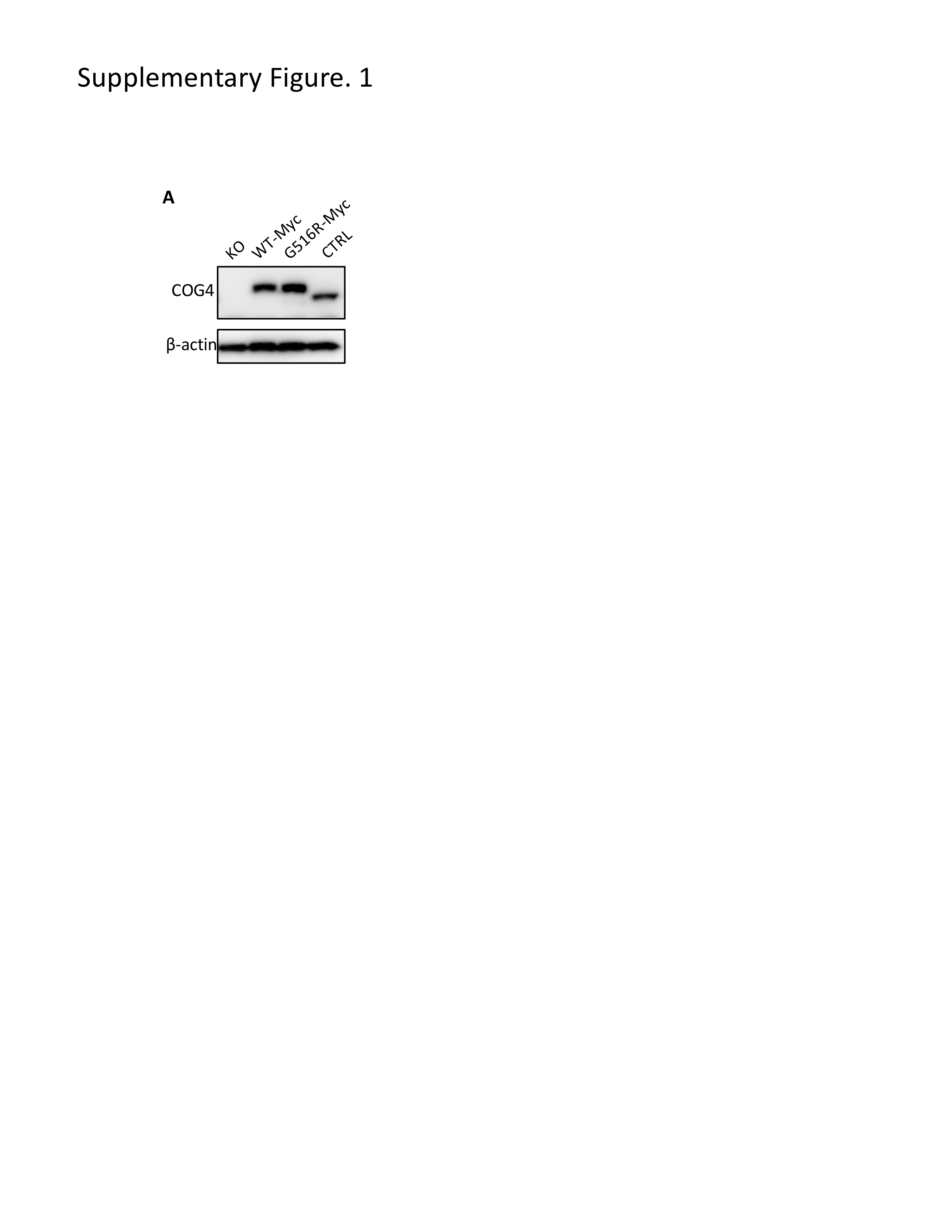

Supplement: Supplementary file 2 [file Image1.JPEG]

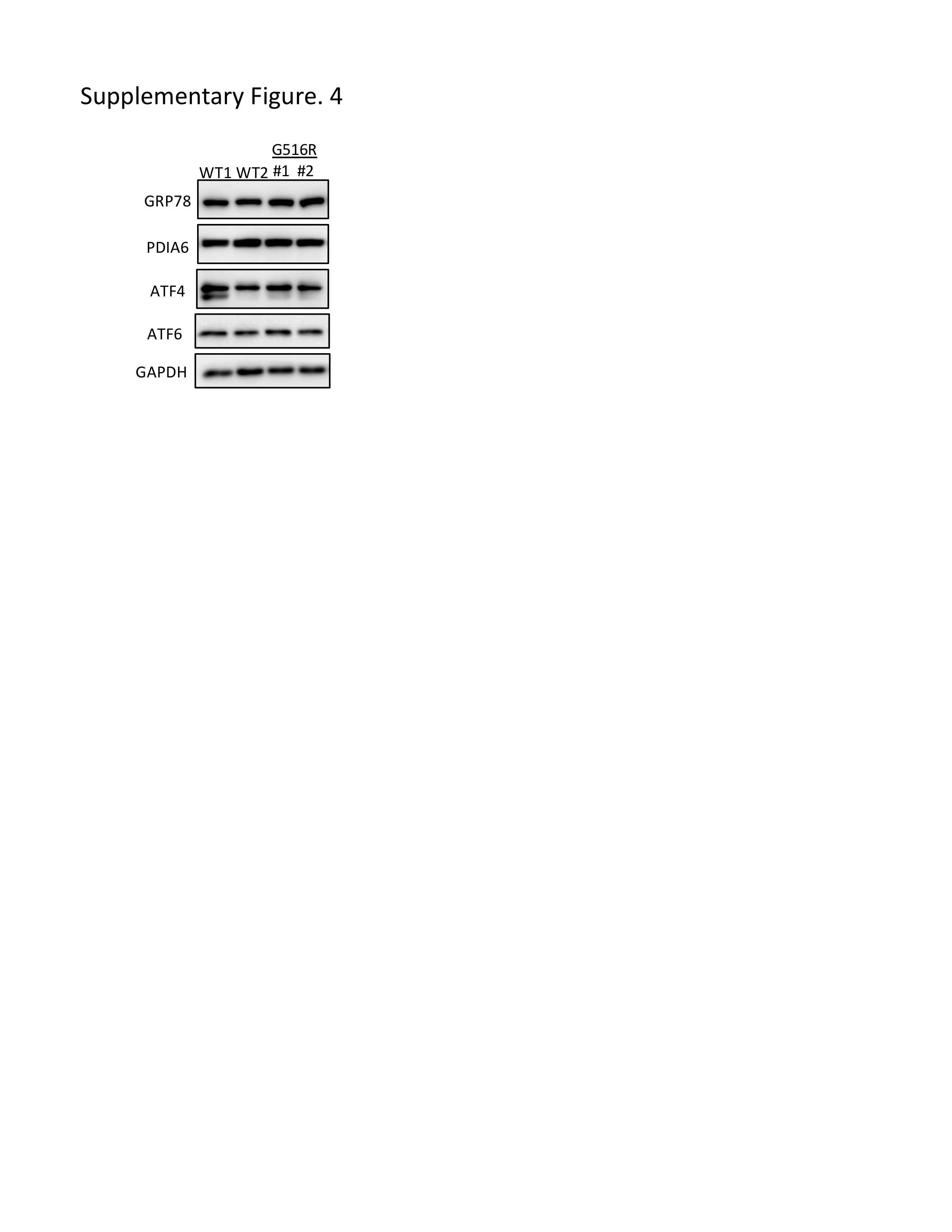

Supplement: Supplementary file 3 [file Image4.JPEG]

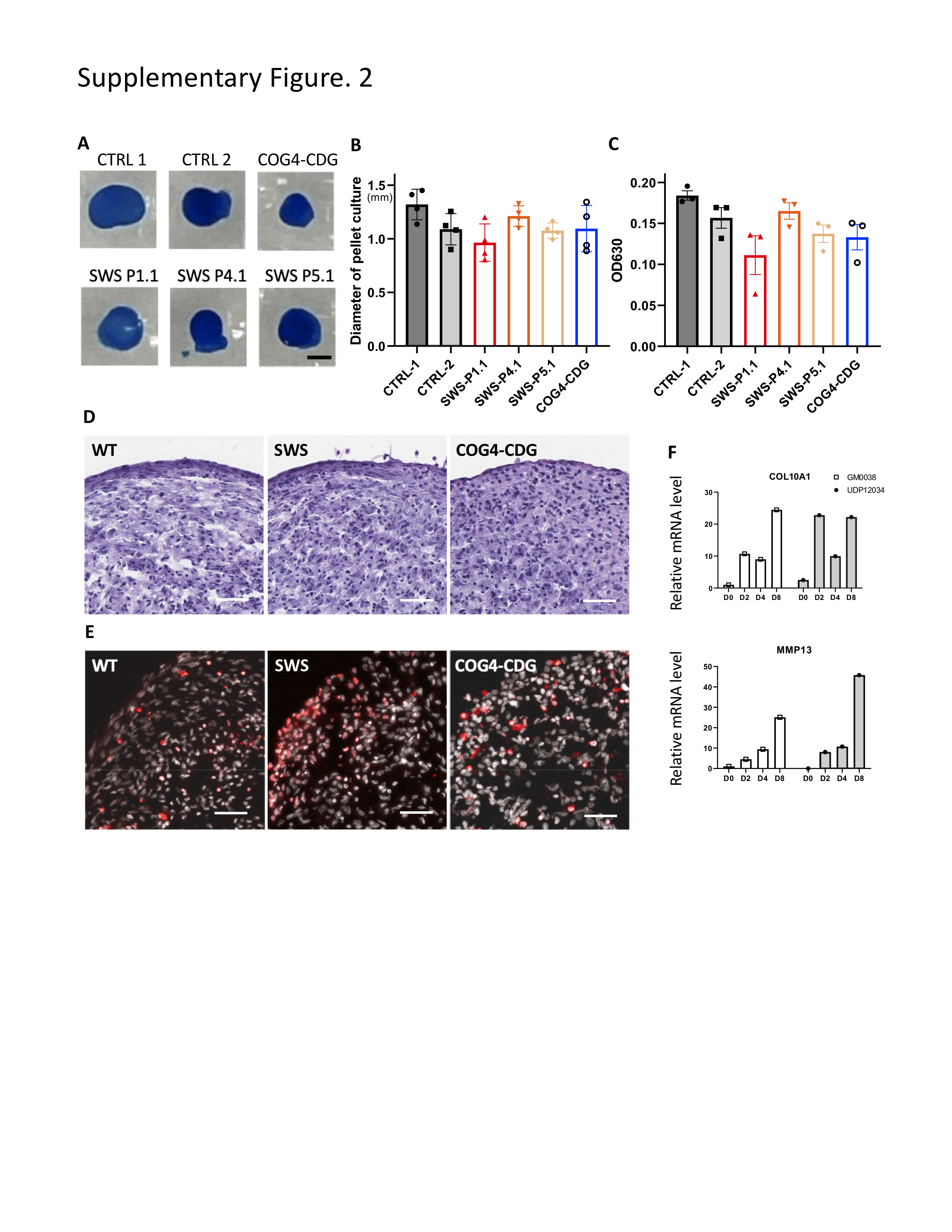

Supplement: Supplementary file 4 [file Image2.JPEG]

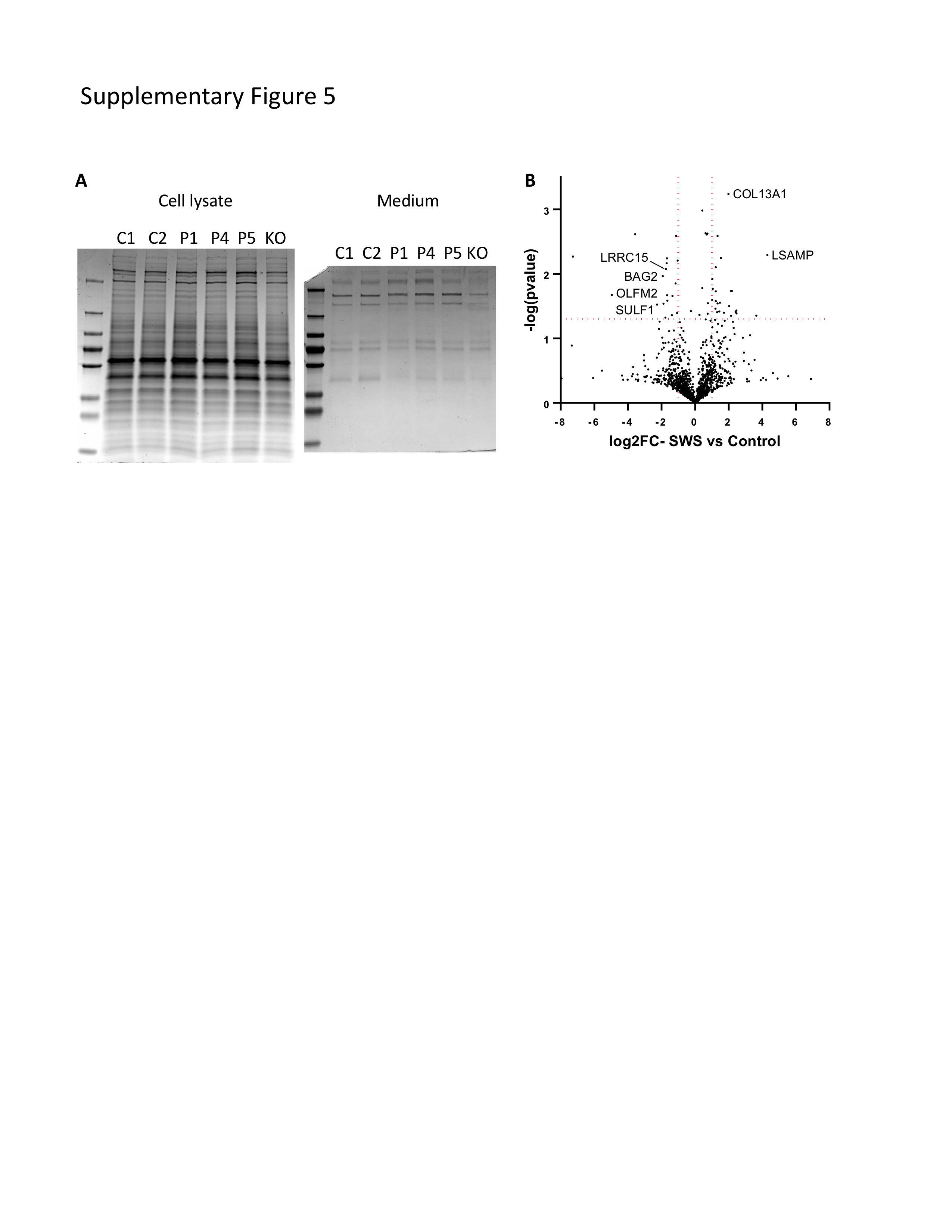

Supplement: Supplementary file 5 [file Image5.JPEG]
